# Nested Bee Hive: A Conceptual Multilayer Architecture for 6G in Futuristic Sustainable Smart Cities

**DOI:** 10.3390/s22165950

**Published:** 2022-08-09

**Authors:** Muhammad Shoaib Farooq, Rana Muhammad Nadir, Furqan Rustam, Soojung Hur, Yongwan Park, Imran Ashraf

**Affiliations:** 1 Department of Computer Science, University of Management and Technology, Lahore 54000, Pakistan; 2 Department of Software Engineering, School of Systems and Technology, University of Management and Technology, Lahore 54000, Pakistan; 3 Department of Information and Communication Engineering, Yeungnam University, Gyeongsan-si 38541, Korea

**Keywords:** 6G communication network, Internet of Things, futuristic smart city, bee hive, multilayer architecture, network slicing

## Abstract

Several smart city ideas are introduced to manage various problems caused by overpopulation, but the futuristic smart city is a concept based on dense and artificial-intelligence-centric cities. Thus, massive device connectivity with huge data traffic is expected in the future where communication networks are expected to provide ubiquity, high quality of service, and on-demand content for a large number of interconnected devices. The sixth-generation (6G) network is considered the problem-solving network of futuristic cities, with huge bandwidth and low latency. The expected 6G of the radio access network is based on terahertz (THz) waves with the capability of carrying up to one terabit per second (Tbps). THz waves have the capability of carrying a large amount of data but these waves have several drawbacks, such as short-range and atmospheric attenuation. Hence, these problems can introduce complications and hamper the performance of the 6G network. This study envisions futuristic smart cities using 6G and proposes a conceptual terrestrial network (TN) architecture for 6G. The nested Bee Hive is a scalable multilayer architecture designed to meet the needs of futuristic smart cities. Moreover, we designed the multilayer network infrastructure while considering the expectations from a network of futuristic smart cities and the complications of THz waves. Extensive simulations are performed using different pathfinding algorithms in the 3D multilayer domain to evaluate the performance of the proposed architecture and set the dynamics of futuristic communication of 6G.

## 1. Introduction

With the rapid increase in urbanization, the frequency and intensity of several problems have been elevated over the past decade. Massive urbanization comes with a lot of problems, such as communication network needs, traffic congestion, parking shortage, massive waste, pollution, and other management and environmental problems [1]. The trend of urbanization gave birth to the idea of a smart city. The concept of the smart city aims at solving such problems using a wide network of a large number of interconnected devices. The smart city is not only a one-directional spectrum; multiple smart ideas grow under the umbrella of smart cities, such as smart grid, smart parking, smart cleaning, smart air quality monitoring, and autonomous vehicles. The smart city is a spectrum that embodies multiple solutions to solve problems caused by overpopulation in urban areas.

The concept of futuristic smart cities is more than just a solution to failures caused by overpopulation. The futuristic smart city is an idea of the smart city 2.0, with artificial intelligence (AI) and ultra-massive urbanization. The world’s population is on a continuous track of urbanization; in 2050, around 68% of the world’s population is foreseen in urban areas [2]. The umbrella of the smart city might not be enough to cover all problems of ultra-massive urbanization; some extra features such as AI and intelligent networks might be required to cover the gap. Several smart ideas have been already introduced for the smart city environment, but these ideas can be upgraded with the spice of AI in futuristic smart cities. For example, AI-based surveillance and smart policing mechanism, autonomous flying car management mechanisms, multi-camera ultra-low-latency live hologram, ultra-low-latency remote surgery, and other intelligent ideas could be introduced. From a little finger ring to large skyscrapers, each and everything is expected to be partially or fully smart.

Futuristic smart cities are not just Internet of Things (IoT)-network-based cities; in the futuristic term, IoT will be changed to the Internet of Everything (IoE), and even human–machine intellectual association is expected through different smart devices [3,4]. Thus, the IoE can bring massive traffic and huge data transfer requirement to the network. In 2030, machine-to-machine (M2M) data usage is expected to be 4394 exabytes (EB), while the use by other domains is anticipated at around 5016 EB; hence, today’s network needs to be upgraded to meet the needs of the future [5]. Networks of futuristic smart cities should have a huge bandwidth, low latency, and AI integration. To implement the smart city idea, smart devices require a communication link to communicate with other devices. The 5G network is a good communication channel to step forward in the smart cities domain. Future data transfer requirements are beyond the capacity of 5G, especially for critical situations such as remote human surgery and other critical AI-based applications where 5G would not be sufficient due to its bandwidth and latency limitations [6,7].

The 6G network is a promising wireless technology to manage the dense connectivity of futuristic AI-centric smart cities. The concept of the 6G wireless network involves ultra-low latency of up to 0.1 milliseconds (ms) and high bandwidth of up to 1 Tbps [7]. Wired networks with fiber optics have a high data transfer rate, but they cannot meet the demand of the futuristic smart city because of mobility constraints. For example, an autonomous vehicle, a moving robot, a drone, and other moving objects cannot be connected with a wire. There is a need for high-bandwidth wireless data connection to ensure mobility. The 6G is expected to be ten times denser than 5G, which makes up to 107 devices per square kilometer [8]. The massive connectivity of smart devices in futuristic smart cities requires cloud infrastructure in a 6G network for data handling and AI computation. The 6G comes with a lot of perks, but it has some implementation challenges, such as the short range of THz electromagnetic waves and their absorption in earth moisture. Elements such as rain, fog, and snow can severely affect the coverage of 6G [9]. A massive infrastructure of the communication network is required for the implementation of 6G.

From this perspective, this study proposes a terrestrial communication network of 6G for futuristic smart cities. The nested Bee Hive is a scalable multilayer architecture designed to meet the needs of futuristic smart cities. The distributed multilayer cloud infrastructure of Bee Hive provides an on-door cloud facility, which makes AI implementation easy and provides low latency for data transmission between cloud and smart devices. Moreover, the design of Bee Hive is formulated while considering the pros and cons of THz waves, and the massive infrastructure of future smart cities.

The rest of the paper is organized as follows: In Section 2, related work for the 6G domain is discussed. Section 3 contains the complications of the THz spectrum and possible challenges for 6G. Section 4 consists of the proposed 6G architecture for smart cities. In Section 5, the communication processes of Bee Hive architecture and algorithms are presented. In Section 6, results and discussions are given, including the evaluation of different algorithms in multilayer space. Moreover, probable obstacles for the 6G network and future directions are elaborated. In the last section, the paper is concluded.

## 2. Related Work

The 6G research is in its initial phases; a long journey is ahead to reach maturity. For now, researchers are just predicting the perks of the futuristic heaven of smart cities and 6G.

For instance, Zhang et al. describe the outcomes of 6G implementation in mobile broadband, AI, and super IoT by analyzing the future needs in these fields [10]. Alsharif et al. also present the visions and key features of the futuristic 6G network [11]. Furthermore, the authors provide the timeline of the 6G evolution and discuss the complications of the THz spectrum. However, not every model architecture is given, which combines all inspirations and visions in the form of a network.

Along the same lines, Ref. [12] paint a broad picture of the futuristic 6G network with a unified experience of the digital and biological world. Moreover, the study discusses the latency lags of 5G which is covered by 6G, and gives a glimpse of possible 6G infrastructure with sub-network and multipath connectivity. Similarly, Ref. [13] discuss massive data usage of the future and envision the possible features of 6G, such as AI support, quantum communication, 3D network, and cell-free communication, which might be part of 6G or may help to shape up 6G infrastructure. However, these studies simply discuss the probable features of the future-envisioned 6G network and lack defining a model network architecture to shape these features.

The authors illustrate the picture of a possible green 6G network, with benefits of the new THz spectrum and visible light communication (VLC), in [14]. Besides the spectrum, different concepts, such as quantum communication, security through blockchain, and ground-to-space networks are also discussed as part of the 6G ambition. Yang et al. [15] present the idea of an architecture similar to green 6G with massive AI using edge and ground-to-space connectivity. However, these works ignore the complications and ground realities of THz waves and do not provide a model network architecture.

Zhang et al. present visions and large dimensional 6G architecture with space, ground, and underwater connectivity in [8]. Furthermore, the study discusses the specifications of 4G, 5G, and 6G for differentiation and the identification of pros and cons. The authors discuss the 6G infrastructure with space and underwater connectivity [16]. However, short-range, atmospheric attenuation, and water influencing factors of THz waves are not considered while designing the architecture. The 6G is under the radar of fame due to its huge bit-carrying capacity but too many expectations cannot be more than fantasy.

Ref. [17] discusses the ubiquitous connectivity of 6G in smart cities with ultra-low latency, ultra-high bandwidth, ultra-high reliability, and super-high intelligence. Moreover, cloud infrastructure with network slicing and space connectivity is also part of the research. However, THz electromagnetic spectrum is the foundation of 6G, which is not even discussed in the research, and the authors build the 6G castle in the air without the foundation.

A small handful of research work is available on 6G, most of which simply discusses its abundant applications with respect to future smart cities. Furthermore, the researchers who discuss the possible architecture of 6G completely ignore the complications of the THz spectrum. The novelty of this work, apart from providing a vision of 6G, is the proposal of a conceptual terrestrial nested network architecture of 6G for futuristic smart cities. In addition, unlike existing approaches, our concept is based on the ground realities of THz waves. It is an initial step to move toward the dream destination of futuristic sustainable smart cities.

## 3. Challenges of 6G Network

**Figure 1:**
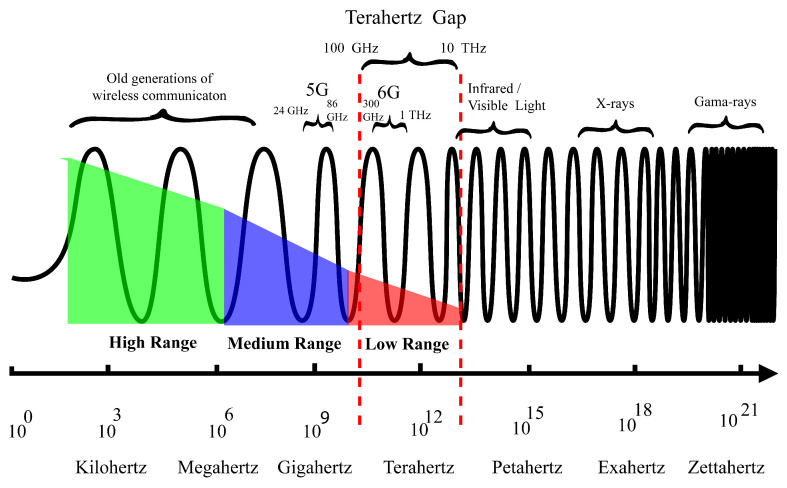
Electromagnetic waves spectrum and range.

The 6G uses THz radiation as a medium of data transfer, which provides more data-carrying capacity due to the short wavelength, but THz waves are not good regarding transmission range, as demonstrated in Figure 1. THz waves have a high absorption rate of Earth’s moisture; water molecules in the air penetrate the terahertz waves and limit their sustainability. Terahertz waves cannot even pass through materials such as walls, glass, and wood, which is a significant part of our constructed infrastructure [11]. The 6G has to face multiple implementation challenges due to short-range and atmospheric attenuation of THz waves. Short range of 6G leads toward the dense connectivity structure, and atmospheric attenuation creates limitations, which makes 6G networking architecture more complex. Apart from short-range and atmospheric allergies, 6G has some general challenges, such as the management of large-scale connectivity of smart devices and their security. This section discusses the major challenges that the 6G network has to face due to futuristic expectations and convolutions of THz waves.

### 3.1. Quality of Service

Quality of service (QoS) is the biggest challenge for the 6G network. Multiple factors may slow down or shut down the 6G link, which may lead to bad quality of service and quality of experience (QoE) for the customer, as users pay highly for higher QoE.

The 6G works on THz waves, which is the biggest hindrance due to the short range of up to 10 m. Service antenna cell is far shorter than 4G and even 5G; only those who are within the ten-meter radius of a 6G antenna can be entertained by the service of 6G due to atmospheric attenuation. The 5G has a range of around 150 m, and THz waves’ attenuation rate makes the establishment of the 6G network more dense and complex. A massive antenna structure is required for the implementation of the 6G network. In 6G wireless THz antenna-to-antenna interlink, every antenna should be at a distance of around 32 feet without any disturbance between them, but fiber optics link can extend the distance between antennas to up to 64 feet. A massive antenna structure is required around the streets of the city due to the short range of THz waves.

Short-range signals are not the only problem for 6G; these signals have a high attenuation level and cannot pass through walls and other city infrastructure [18]. This characteristic of 6G makes it more challenging to implement in the structure of the smart city with buildings and massive infrastructure. If these signals cannot pass through the walls, then a separate indoor network for houses and other smart buildings is required. Moist weather is the biggest challenge in the implementation of the 6G network. If it is raining outside, then 6G may face signal drop or low speed, and in the worst case, may completely drop the link. In the futuristic smart city, fog, heavy rain, or storm can destroy the QoS of the network.

### 3.2. Quality of Experience

QoS is worthless in the futuristic environment if the network is unable to provide QoE. A smart monitoring system is required which identifies the user needs, by following the patterns of collected data from different smart devices. Dense device connectivity is expected in the 6G network, which will generate massive data. Thus, the 6G network should have huge data storage capacity and computation power to identify data patterns, which helps mobile network operators (MNO) to provide a better experience to the customers.

### 3.3. AI-Based Network

AI is the core part of 6G; different patterns could be designed based on the data collected from smart devices. Numerous AI models could be introduced to manage the network and deal with various issues. Multiple cyberphysical systems can be part of the smart city, such as robot delivery and autonomous cars. These systems might be moving continuously from one cell to another cell; there should be an intelligent network to maintain a connection with them.

### 3.4. Security

The IoE connects everything to the network; one thing that comes to mind is the security of such networks. Security is always a challenge for every system. Adversaries can misuse the data collected from a smart device or can breach privacy by hacking the network. Security is a very important measure that cannot be ignored in the 6G network because 6G connects all smart devices carrying personal and confidential information. Not only the general public but also different law enforcement agencies and government departments can be part of the 6G network, and one breach can cause severe damage.

## 4. Proposed Nested Bee Hive Architecture

The nested Bee Hive is the proposed conceptual architecture that illustrates the physical structure of 6G for futuristic smart cities. Nested hive deals with 6G and previous generations in the form of nested layered hives. First, the city is divided into cells of 3 to 10 miles radius for optimal 4G coverage [19,20,21]. The area of the cells may be set with respect to the density of connected devices or scaling up the resources in that cell. The number of cells depends on the area of the city, each cell is called a town cell, and each cell has its local AI-based cell station. Each cell station is linked with its neighboring cell station through a fiber backhaul link. The city gateway (CG) is the base station of the city, which connects the city network with other city networks and the internet.

An area of 0.6 square miles may contain around ten million devices in smart cities of 2030 [8], which justifies the use of AI-based organization of resources on the edge to reduce the congestion on the higher levels of the network. A 3- to 10-mile radius town cell seems too small an area distribution, and it is not fixed, but it may contain more devices than today’s megacity. A lot of AI-based smart devices are expected to intercommunicate with other smart devices and cloud services. A massive load of smart devices can create trouble for existing network infrastructure. Bee Hive architecture is based on an edge computing concept that deals with local traffic within the cell. Cell stations, smart distribution unit (SDU) and ultra-smart distribution unit (USDU) are AI-based stations that work as local network controllers in their domain.

**Figure 2:**
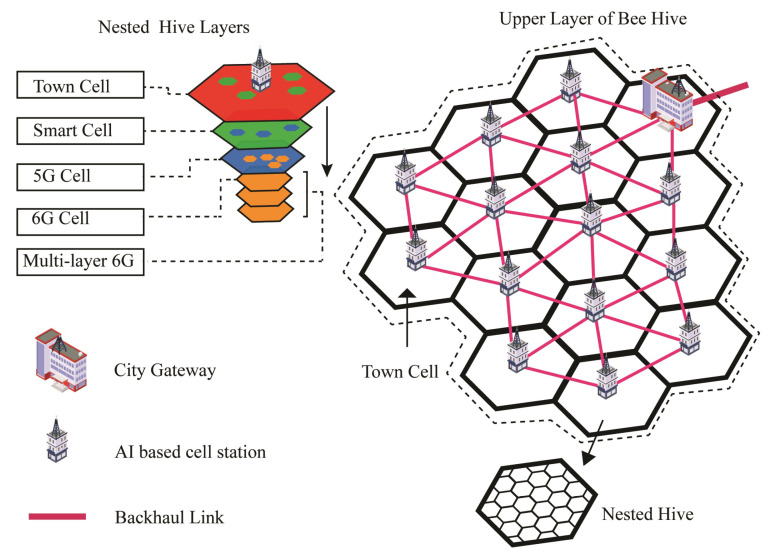
Nested cells distribution view of Bee Hive architecture.

Figure 2 illustrates the nested layers of Bee Hive architecture with multiple town cells and interconnectivity. Communication with other town cells is through the cell station of that specific cell, which is connected through fiber links. The path selection between cell stations can be through the shortest path, low-traffic path, or other AI-based algorithms. The CG station is the primary network controller of the city, which connects the city with the Internet and other smart cities. CG also synchronizes the data from its child clouds or town cell clouds to its central cloud. Fiber backhaul links are suitable for connectivity between cell stations due to bandwidth and needs of the future. The 6G and 5G backhauls cannot be used due to their short range and atmospheric attenuation. The 4G backhaul can be used at this distance, but it is a low-bandwidth spectrum. However, a 4G tower could be installed in the town cell station to provide 4G coverage in the town cell.

The upper layer of Bee Hive architecture containing a city gateway and multiple town cells creates a distributed cloud infrastructure, which makes edge computing easy. Distributed AI applications on the town cell cloud can cause low latency and low traffic burden on the upper network [22]. Edge computation might be required for futuristic ideas, such as antonyms flying cars, smart police, and other cyberphysical systems (CPS) concepts. AI algorithms require a lot of computing power; it is challenging for a small smart device to run heavy-duty algorithms. The edge computing concept empowers the local cloud partially run the algorithm, which reduces the burden from small smart devices and makes them easy to operate. Furthermore, Bee Hive provides security to different classes of connected devices by isolation of one class from another through network slicing.

**Figure 3:**
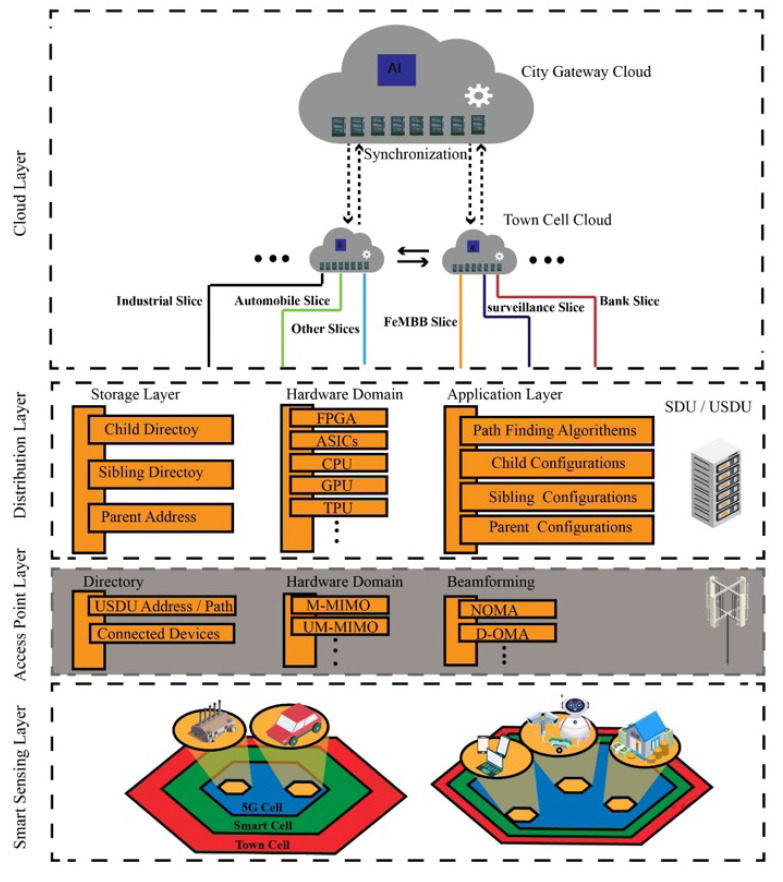
Logical layers in Bee Hive architecture.

Figure 3 demonstrates the logical layers of Bee Hive architecture. The smart sensing layer or data collection layer consists of multiple devices and sensors in 6G cells. The access point layer is a basic communication layer of the network which connects devices in the sensing layer with the network. Access point keeps the address of connected devices, USDU, and neighbor access points in the directory. For the hardware domain of access point, massive multiple input and multiple output (M-MIMO) and ultra-massive multiple input and multiple output (UM-MIMO) are used, but other hardware with massive input and output capability can also be used. The access point of the 6G cell uses beamforming to access the connected devices, non-orthogonal multiple access (NOMA), delta-orthogonal multiple access (D-OMA), or any other beamforming protocol can be used according to the need in the cell.

The distribution layer encapsulates SDU and USDU. SDU and USDU have the same properties but work in different domains. The USDU controls the distribution within a 6G hive while SDU controls the distribution of USDUs in smart cells. The storage layer of the distribution layer maintains the directory of siblings, child, and parent. The application layer of the distribution layer consists of parent, child, and sibling configurations. Furthermore, pathfinding algorithms such as directional traverse, directional greed, 3D Dijkstra, and others also work on the application layer.

The cloud layer in Figure 3 demonstrates the distributed cloud concept of Bee Hive architecture which is similar to the fog architecture of computing; each town cell device communicates with the local town cell cloud in the form of slices, then the town cloud communicates with city gateway cloud (CGC). The city gateway station is a central certification authority (CCA) that certifies all the devices in the city network; otherwise, these devices will be considered alien devices. Connection of an alien device with the network will be denied by town cell station or city gateway, public key infrastructure (PKI), or other cryptographic ways that can be implemented for secure encrypted communication [23].

### 4.1. Nested Hive

Nested hive is a sub-distribution of town cell, it consists of 1-mile radius cells, and each cell is interconnected with fiber or 6G mid-haul. The 6G can be used as a mid-haul between cell station and SDU by implanting an array of 6G antennas on a specific distance, but the fiber link is more reliable in case of high-moisture areas. The SDU contains the directory of the subsidiary network and other SDUs in the town cell. The SDU is the distributor of smart cells and helps in peer-to-peer (P2P) communication by direct communication with other smart cell SDUs through interlink or mid-haul link. Mid haul link is a specific link that connects the SDU to a cell station. On the other hand, an interlink is an array of 6G antenna devices that connect one SDU to another SDU. The cell station is a local cloud and distributor of town cell; on the other side, SDU performs the same services on a small scale of smart cell other than cloud services. There is a huge gap in coverage between 4G and 5G; the fourth generation of the wireless network gives coverage of approximately 10 miles and 5G provides around 500 to 1000 feet, which are not even 2% of the 4G range. The nested hive creates a bridge between 4G cells (town cell) and 5G cells.

**Figure 4:**
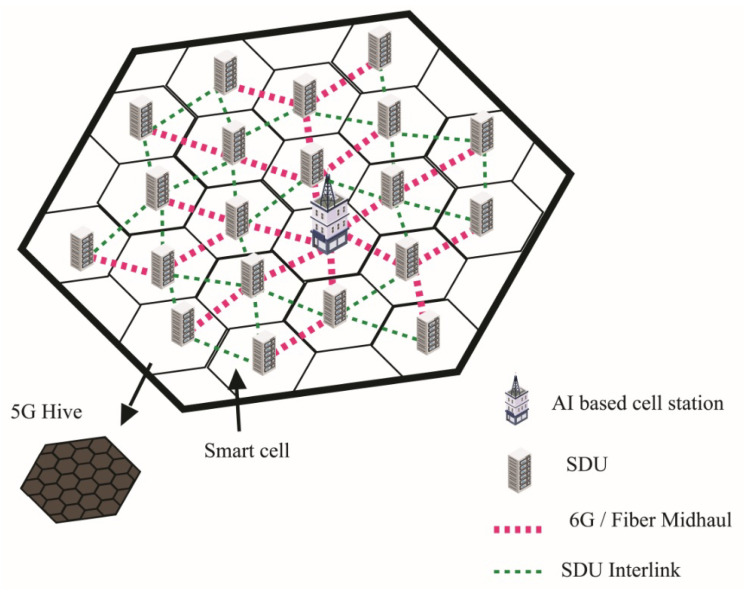
Hive of smart cells.

Figure 4 illustrates the physical structure of the nested hive, with multiple smart cells having SDUs. Bold dotted lines demonstrate that each smart cell SDU is connected with the cell station through a fiber mid-haul link and small dotted lines show a 6G interlink of SDUs.

### 4.2. 5G Hive

A smart cell is distributed in multiple cells up to a 1000 feet radius, which is the core need for better 5G coverage [24,25]. Each cell has its 5G pole and USDU for further distribution of 6G. The USDU works the same as SDU but the domain area is smaller and denser than SDU. Furthermore, 5G pole and USDU are connected to SDU through fiber or 6G front haul link. A 5G front haul or interlink can be used if the further distribution of 6G is not required in that area. A 4G cell tower could be installed in the smart cell in case of dense 4G usage in the town cell.

**Figure 5:**
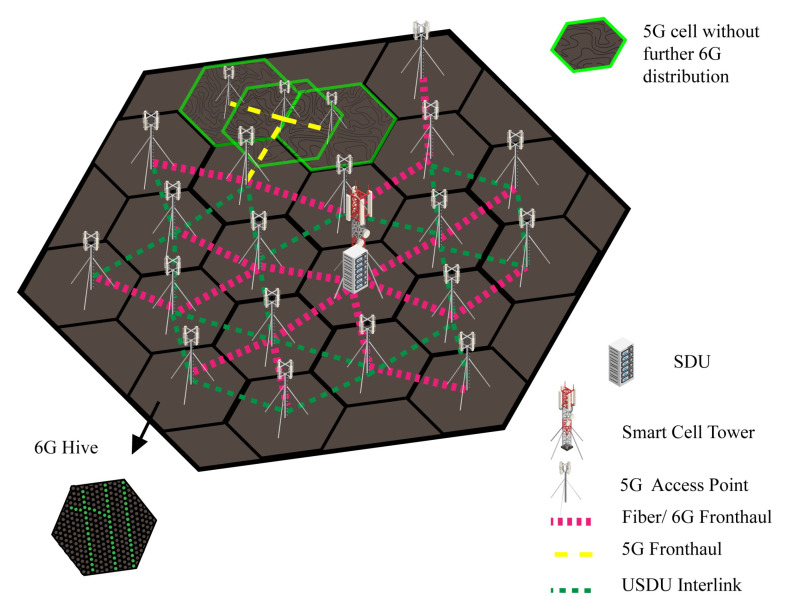
5G cells hive.

Figure 5 projects the picture of a smart cell with multiple 5G cells. Bold dotted lines represent fiber front haul link, small dotted lines represent USDU to USDU 6G interlink, and the long dashed line represents 5G interlink. The 5G interlink can be used in the case of no further distribution of 6G, and it can create an overlapping cell, as demonstrated in the figure.

### 4.3. 6G Hive

**Figure 6:**
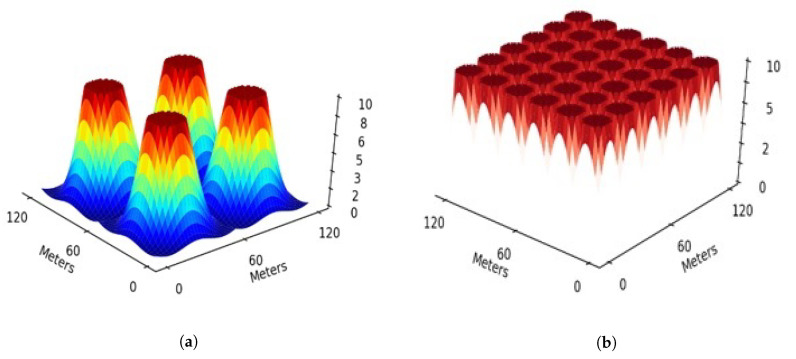
Spectral efficiency of 6G, (**a**) Spectral efficiency;10 is the peak efficiency, *x* and *y* axes shows area in meter, and (**b**) Efficiency using multiple access points of 6G.

The 6G hive is a subset of the 5G cell, and each 6G cell has a spectral radius of around 10 m, which drops spectral efficiency rapidly after reaching the range. Figure 6 illustrates the spectral efficiency and coverage area of the 6G access point or node. Diagonal scales show the area in meters, and the value 10 demonstrates the peak efficiency in the vertical scale, which descends immediately after crossing the spectral border in Figure 6a. Figure 6b consists of multiple 6G access points which keep the spectral efficiency on peak and prevent the fall in spectral efficiency.

Sixth generation coverage cannot be provided in the entire 5G cell due to its short range and buildings infrastructure of the smart city. For this, the 6G hive is distributed in two parts, active and inactive cells. Active cells are the real coverage area of 6G. The proposed method can only be achieved through the road and street infrastructure of the city because 6G signals are short-range and cannot pass through walls. Street light poles and roadside objects are ideal for the implementation of 6G infrastructure; the distance between each pole depends on interlinking either using fiber or 6G. An array of 6G interlinks/access points is required for a site without the requirement of continuous service in an inactive area of the 6G hive, which requires 6G to operate robots or devices. Remote areas without 6G coverage can be connected to any nearest 6G hive through fiber link. For the internal structure of buildings, each building requires its 6G access antenna. Inactive cells might be the area covered by building or free area where 6G coverage is not required, but coverage in inactive cells can be achieved through an unnamed aerial vehicle (UAV) network due to AI computation power in the town cell cloud, which makes 6G scalable.

**Figure 7:**
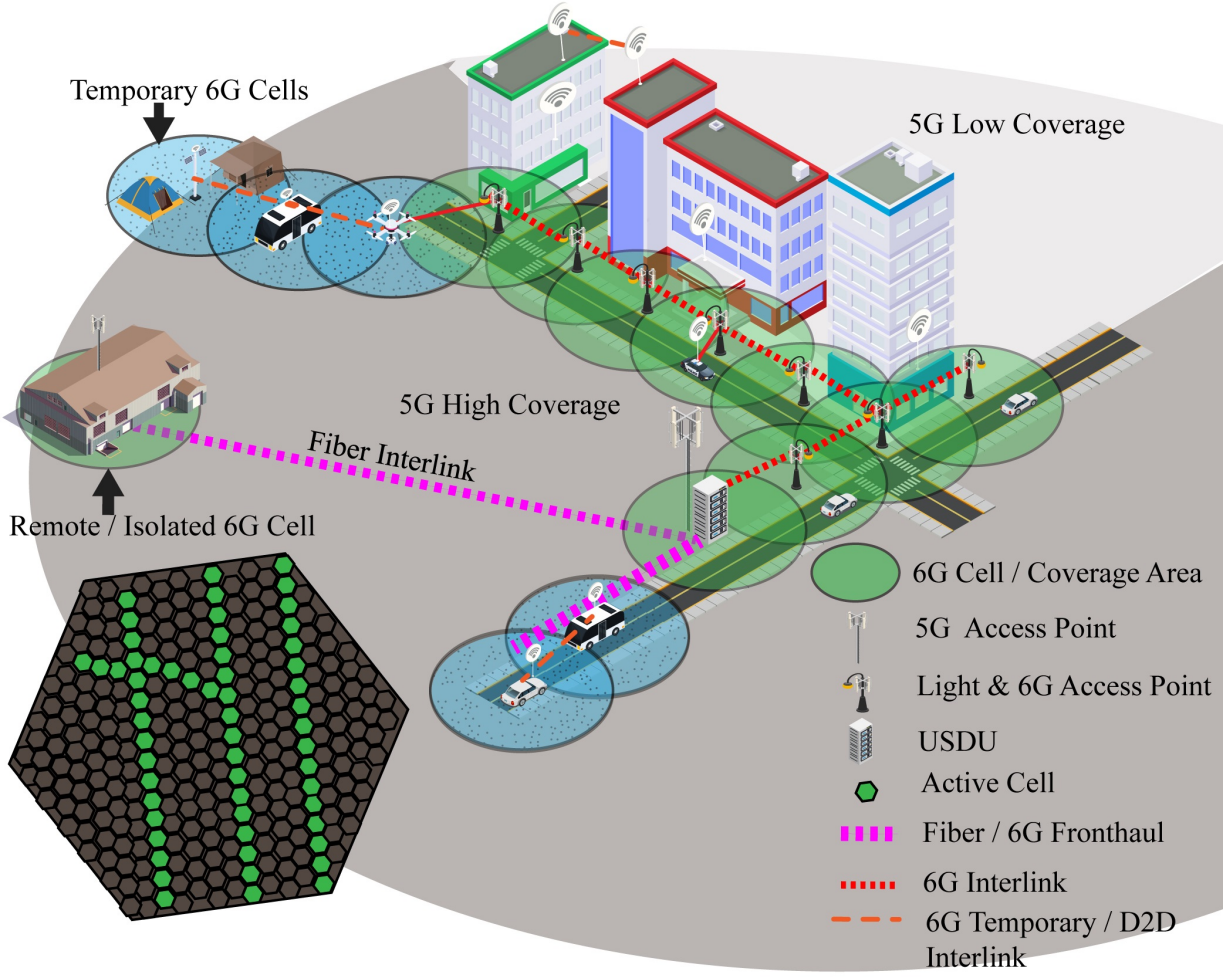
Structure of 6G hive.

Figure 7 illustrates the initial structure of the 6G infrastructure. The big circle around the buildings represents a 5G cell, the dark shaded area represents high coverage area, and the light shade represents a low- or no-coverage area of 5G due to interruption of buildings. The area with disturbed coverage can be covered through extra access point antennas in the cell. Small circles on the road represent the coverage area of 6G, light poles are used as 6G poles due to the short range of 6G, and, further, these light poles can be used for VLC or light fidelity (LIFI) in futuristic smart cities. Temporary or device-to-device (D2D) 6G interlink is represented with dashed line accomplished through UAV and smart 6G vehicle, which can be used to provide 6G service in a non-6G area. The D2D 6G interlink could be private or public depending on the requirement. An isolated 6G cell, as shown in Figure 7, connects with USDU fiber interlink; it can be also used to provide 6G service to a remote facility. The USDU is the local distributor and controller of the cell network which contains the directory of access points and interlink paths in the cell. Furthermore, it contains the directory of other USDUs, interlink paths, front haul paths, and parent SDUs.

### 4.4. Multilayer 6G

The proposed system exists in 3D space; the 6G range is not only short in *x* and *y* dimensions but also the *z* dimension. Flying objects such as drones and UAVs are considered an essential part of futuristic societies; thus, these UAVs require real-time communication links to perform different functions and avoid collision. Visions of flying objects with THz communication links in the shadows of skyscrapers cannot be achieved through the ground layer of the network, because 6G has a short range and it cannot cover the height of huge buildings, thus multilayer 6G network is needed to cover the upper area. Bee Hive provides a multilayer network to raise the aroma of 6G in the air of futuristic smart cities, and multidirectional interlinking of small access points can help to create the cubic or mesh net of 6G.

**Figure 8:**
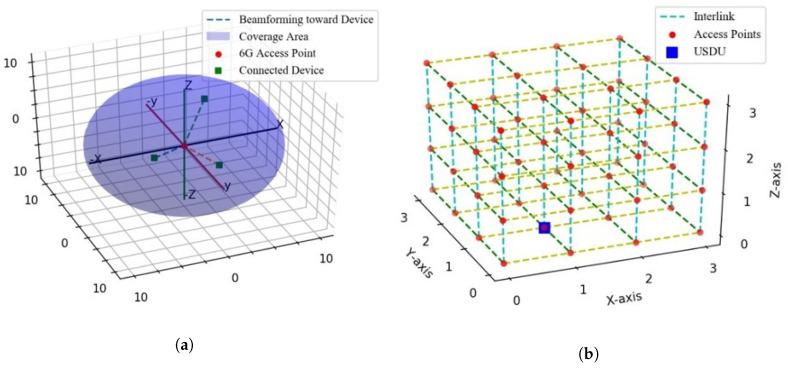
Three-dimensional multilayer network connectivity. (**a**) Coverage of 6G access point in *x*, *y*, and *z*, and (**b**) 3D connectivity of multiple 6G access points.

**Figure 9:**
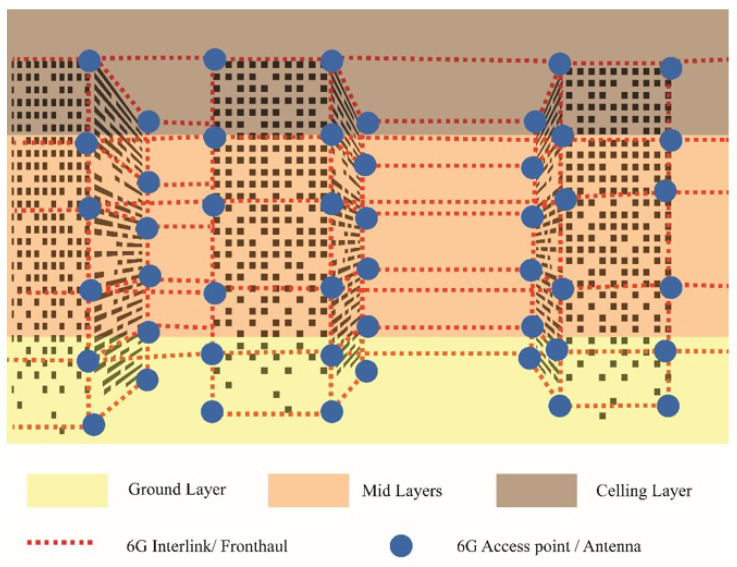
Multilayer networks connectivity in futuristic cities.

Figure 8 demonstrates the multilayer network connectivity of Bee Hive. Figure 8b shows the coverage of a 6G access point in *x*, *y*, and *z* dimensions while Figure 8a illustrates the 3D connectivity of multiple 6G access points, their cubic interlink, and their location in the 3D world. Figure 9 encapsulates a glimpse of the smart 6G network in futuristic smart cities. The ground layer is a normal 6G hive layer as discussed before; the ceiling layer defines the height limit of the 6G network and the mid-layer consists of multiple layers depending on coverage height. Round spots in Figure 8 are the access points of 6G, and dotted lines represent the 6G interlink or antenna-to-antenna communication.

### 4.5. Indoor 6G

Sixth generation can be more popular for indoor networks. THz waves have many challenges to face outdoor, such as moisture in the atmosphere and short range, but in indoor computing, it may be revolutionary for communication between devices. In data centers, a lot of devices and servers are interconnected with each other through the wired link, due to heavy communication between servers and devices. In the future, a 6G interlink could be used between servers and devices for communication due to its high bandwidth and low latency. In the envisioned future, robotics and AI are major players, but wired network limits their use in the sense of mobility of robotic objects. Robots taking care of data centers require good communication links, but if they are wired with fiber links, it can limit their mobility. The 6G can also help in the industrial domain; heavy-duty giant robots are used for manufacturing in various industries. To expand the spectrum of the industrial domain, industries need smart robots for production and quality control, which creates the need for intercommunication of robots. Fiber link limits mobility, and existing recent radio network systems have limitations such as low bandwidth and latency, but 6G ensures high bandwidth and low latency and mobility in the device-to-device (D2D) communication.

## 5. Communication Process

The communication structure of Bee Hive architecture handles a significant chunk of traffic on the ground level, which reduces the traffic load on the upper layers of the network. Bee Hive architecture is an accurate picture of mobile edge computing (MEC); resources deployed on edges such as USDU and SDU handle non-cloud communication within the town cell without putting the load on the town cell station. Requests are handed over to cell stations are cloud services requests, internet requests, and requests for other town cells.

**Figure 10:**
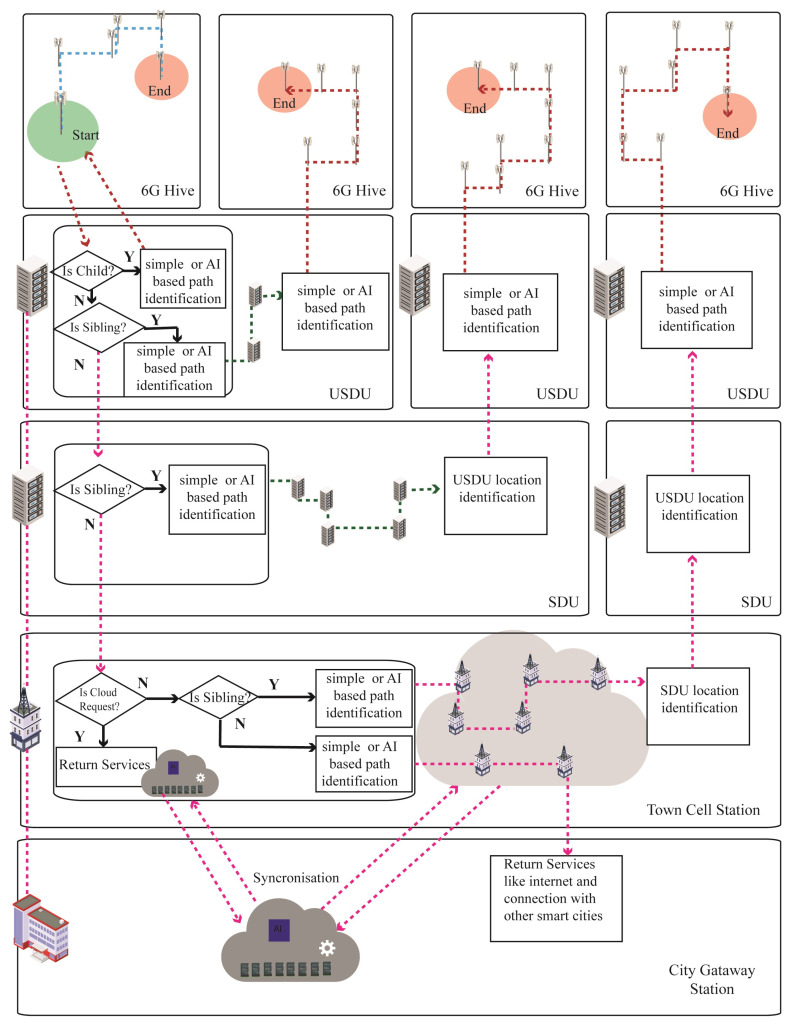
Communication process of 6G hive.

Figure 10 explains the communication process of Bee Hive architecture. Requests generated by the device in the range of any access point of 6G are directly sent to the USDU by a predefined interlink path. The USDU checks if the destination is in its child domain then the distribution unit sends the path of the destination to the sender for direct connection. If the destination is not a child’s location then the USDU checks if the address is a sibling. If yes, the USDU will find the path of the sibling USDU and send the request to it. The sibling USDU finds the path of the destination access point and device to create the connection. Moreover, if the USDU identifies the destination as non-sibling, it will identify it as a communication request from 5G devices within the 5G cell, then it will be sent to the 5G access point; otherwise, it will send the request to the SDU.

The SDU applies the sibling check; if yes, then the path of that particular sibling is identified, and the request will be sent to it. The sibling SDU identifies the destination USDU and sends the request; then the destination USDU identifies the path of the relevant access point and device to make a connection. On the other side, if the SDU identifies the address as a non-sibling, the SDU sends it to the cell station.

The cell station applies the cloud request test first, and if the request is found positive for cloud service, the cell station returns cloud services to the request initiator. However, if the request is not for cloud services, then the cell station applies the sibling test and sends it to its destination sibling after path identification. All non-sibling requests will be identified as communication requests from 4G devices within the town cell, then requests will be sent to 4G access point, otherwise automatically sent to gateway station and connection with other smart cities.

### 5.1. Communication within 6G Hive

The USDU is the central controller of communication within the 6G hive, and is also responsible for incoming and outgoing communication in the hive. The 6G access point or node sends the connection request to the USDU, then Algorithm 1 executes and returns the path in case of connection within the hive. The pathfinding module is pluggable and any pathfinding algorithm can be used, which works on the 3D connectivity of nodes in the network.
**Algorithm 1** USDU connection request.**Input:** specific request for connection or service**Initializations:** Start_state = requesting state          destination_state = get_destination()          // returns the destination node or state 1:**if** (destination_state == Child) **then** 2:   // call specific path finding function 3:   Path, Cost=3D-DirectionalTraverse(start_state,destination_state)   Or 4:   Path, Cost=3D-DirectionalGreed(start_state,destination_state)   Or 5:   Path, Cost=3D-Dijkstra(start_state,destination_state)   Or 6:   // Any pluggable simple or AI-based pathfinding algorithm 7:**else if** (destination_state == Sibling) **then** 8:   // send the request to sibling after finding destination USDU 9:**else if** (destination_state == 5G device within 5G cell/6G Hive) **then**10:   // send the request to the 5G access point of the same cell11:**else**12:   //Send the request to parent SDU13:   // Parent SDU(request)14:**end if**15:Return path/connection or service

#### 5.1.1. 3D-Directional Traverse

3D-directional traverse is an algorithm that moves unidirectional at a time in *x*, *y*, or *z* dimension; if the algorithm moves in *x* dimension, it reaches the level of destination in *x* dimension then it goes for other dimensions. A unidirectional move gives six possible combinations of paths with the same amount of hops, from which the path with minimum cost can be selected. The 3D-directional traverse limits the paths from one to another state which places the ceiling cap on data transfer between two nodes up to 6 Tbps in case of no other traffic on the path. Algorithm 2 is a unidirectional algorithm with *x* dimension first, then *y* and *z*, respectively.

**Figure 11:**
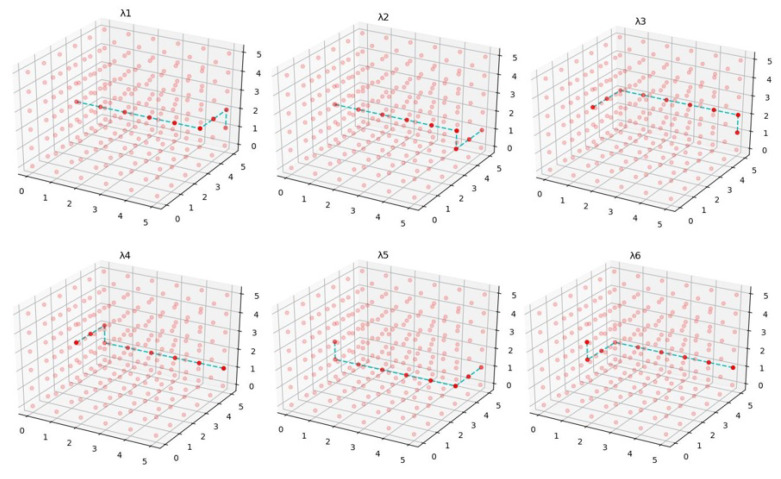
Possible combinations of paths from one state to other particular states.

Figure 11 illustrates six possible combinations of paths from start state (0, 3, 2) to destination state (5, 5, 1). λ1 moves in *x* dimension first then *y* and *z*, respectively. λ2 moves in *x*, *z*, and *y* pattern, λ3 moves in *y*, *x*, and *z* pattern, λ4 moves in *y*, *z*, and *x* pattern, λ5 moves in *z*, *x*, and *y* pattern, and λ6 moves in *z*, *y*, and *x* pattern. A low-cost path from six paths can be extracted using

(1) λp=min(λ1·cost,λ2·cost,λ3·cost,λ4·cost,λ5·cost,λ6·cost).

**Algorithm 2** 3D-directional traverse.
**Input:** start_state, destination_state
**Declarations:** Path=[]                                                                        // list to store path
                        Cost=[]                                                                    // list to store the cost

**Initialization:**

               current_state = start_state
               Path.append(current_state)
 1:**while** (current_state != destination_state) **do** 2:   // x, y, z are the dimensions that collectively make a state or access point 3:   // we can traverse in different dimensions which gives us different results 4:   // current algorithm first travels in *x* dimension first then *y* and *z* 5:   **if** (current_state.x < destination_state.x) **then** 6:     Cost.append(current_state.x_up_cost) 7:     Current_state.x = current_state.x +1   // state moves upward in *x* dimension 8:   **else if** current_state.x > destination_state.x) **then** 9:     Cost.append(current_state.x_down_cost)10:     Current_state.x = current_state.x -1   // state moves downward in *x* dimension11:   **else if** (current_state.y < destination_state.y) **then**12:     Cost.append(current_state.y_up_cost)13:     Current_state.y = current_state.y +1   // state moves upward in *y* dimension14:   **else if** (current_state.y > destination_state.y) **then**15:     Cost.append(current_state.y_down_cost)16:     Current_state.y = current_state.y -1   // state moves downward in *y* dimension17:   **else if** (current_state.z < destination_state.z) **then**18:     Cost.append(current_state.z_up_cost)19:     Current_state.z = current_state.z +1   // state moves upward in *z* dimension20:   **else if** (current_state.z > destination_state.z) **then**21:     Cost.append(current_state.z_down_cost)22:     Current_state.z = current_state.z -1   // state moves downward in *z* dimension23:   **end if**24:   Path.append(current_state)   // send the request to sibling after finding destination USDU25:
**end while**
26:**Return** Path, Cost


#### 5.1.2. 3D-Directional Greed

The 3D-directional greed is an algorithm that moves in a dimension where cost is minimum till the very next node. This algorithm decides on momentary greed that might be more costly in the next hops. With every hop jump, it checks the minimum cost in the direction of the destination. As demonstrated in Algorithm 3, if the destination node *x*’s value is greater than the current node *x* value, then directional greed only checks in *x* up or positive direction cost, not in negative, and the same is true for the *y* and *z* dimensions.
**Algorithm 3** 3D-directional greed.**Input:** start_state, destination_state**Declarations:** Path=[]                                                                                  // list to store path                        Cost=[]                                             // list to store the cost of greed decision**Initialization:**               c_s = start_state                                                                               // current_state               d_s = destination_state               Path.append(c_s) 1:**while** (c_s != destination_state) **do** 2:   // check the status current state by analyzing the x, y, and z dimensions 3:   ( ∧ ) = any condition <, >, = 4:   **if** (c_s.x ∧ d_s.x and c_s.y ∧ d_s.y and c_s.z ∧ d_s.z ) **then** 5:     Cost.append(current_state.x_up_cost) 6:     // in case of < check the down cost of particular dimension 7:     // in case of > check the down cost of particular dimension 8:     // in case of = no need to check dimension it is on destination 9:     Cost.append(min(dimensions))10:     c_s = c_s.trevers(min(dimention))11:     // state moves in(+ or -) x, y or z dimension12:     Path.append(c_s)13:   **end if**14:**end while**15:**Return** Path, Cost

**Figure 12:**
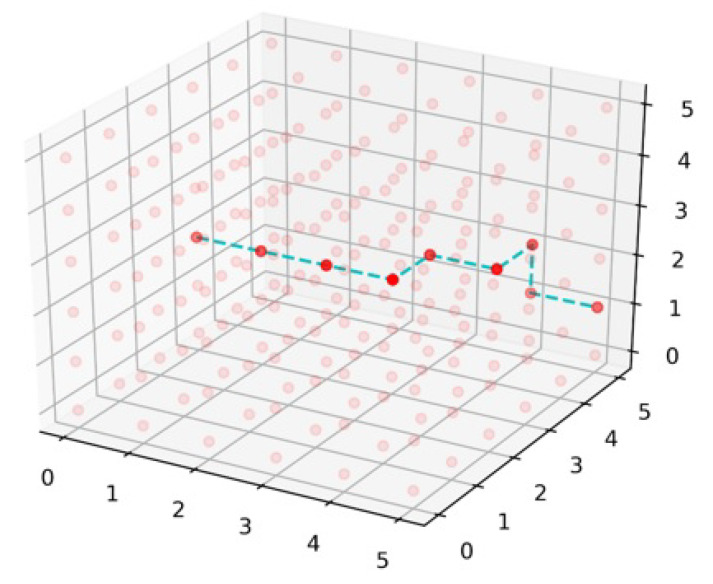
3D-directional greed path.

Figure 12 exemplifies the path from state (0, 3, 2) to end state (5, 5, 1) with directional greed algorithm. First, the algorithm finds the minimum cost in the *x* dimension with every hop till state (3, 3, 2), then it moves in the *y* dimension to state (3, 4, 2) due to the minimum cost hop. From state (3, 4, 2), it moves in *x* dimension then *y* dimension to the state (4, 5, 2), where the algorithm finds –z dimension with the minimum cost then *y* on the next state, which results in the completion of pathfinding with the destination state.

#### 5.1.3. 3D-Dijkstra

**Figure 13:**
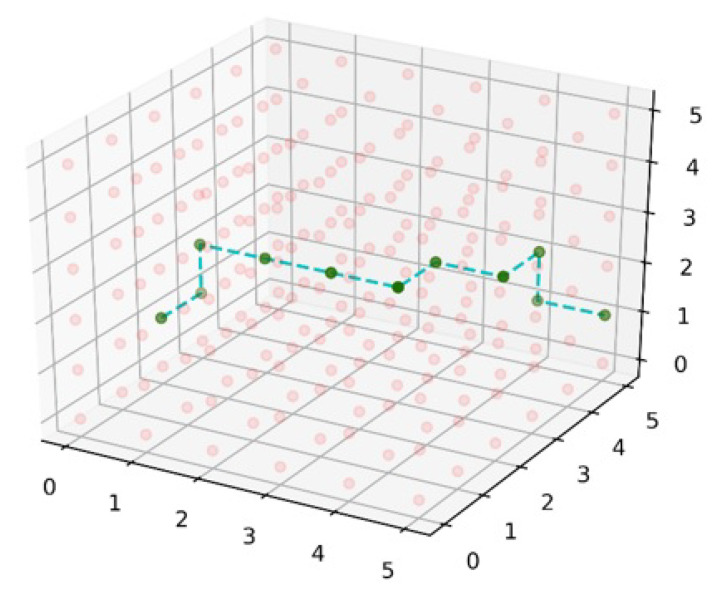
3D-Dijkstra path.

Dijkstra is a widely used algorithm for network pathfinding domain, but the difference between simple and 3D-Dijkstra is the connectivity of nodes in a 3D fashion. In 3D-Dijkstra, each node is connected with six neighbor nodes in *x*, *y*, and *z* dimensions, except boundary nodes of the network. Dijkstra checks the low-cost path from the start to every node in the multilayer network of 6G, which results in the most optimal low-cost path from the start state to the destination state. Dijkstra gives the minimum cost path but the computation cost and time increase due to the calculation of massively connected nodes. Algorithm 4 explains the implementation of 3D-Dijkstra, which takes the input of the start state and destination state and prepares a 3D graph of nodes to implement Dijkstra. The 3D-Dijkstra path is exemplified in Figure 13, where the path starts from state (0, 2, 1) and ends on (5, 5, 1). Dimension *z* is the same in the start state and end state but moves up in the *z* dimension on the state (0, 3, 1) for the sake of low cost.
**Algorithm 4** 3D-Dijkstra.**Input:** start_state, destination_state**Declarations and Initializations:**               graph = get_graph()               Path = []                queue = []                                   // to add graph states               Cost[start_state] = 0 1:**for all** state *i* in graph **do** 2:   **if** state != start_state) **then** 3:     Cost[i] = infinity          // cost of all states other than start state set as infinity 4:     Pred[i] = undefined                    // predecessor state is unknown at this point 5:     queue.append(i) 6:   **end if** 7:   queue.append(i) 8:**end for** 9:**while** queue is not empty **do**10:   Current_state = state in queue with min cost[Current_state]11:   Remove current_state from the queue12:   **for all** neighbor *v* of current_state **do**13:     // directly connected nodes in x, y and z dimension14:   temp = Cost[current_state] + act_cost(current_state,v)   // act_cost is actual cost traversal cost from current state to v state15:   **if** (temp < Cost[current_state]) **then**16:     Cost[v] = temp17:     Pred[v] = current_state18:   **end if**19:  **end for**20:**end while**21:**if** (Pred[destination_state] is defined or destination_state = start_state): **then**22:   **while** destination_state != start_state -1 **do**23:     Cost.append(current_state.x_up_cost)24:     Path.append(destination_state)25:     destination_state=pred[destination_state]26:   **end while**27:**end if**28:**Return** Path, Cost[destination_state]

## 6. Results and Discussions

For the evaluation of different path finding algorithms in a 3D network of 6G hive, simulations are performed in Python 3.7. To evaluate the run-time response of the algorithm, simulations are performed on Hewlett-Packard i5 with 8 GB random access memory (RAM). To give a futuristic touch of computation power, experiments are also executed on the Google computation engine (CoLab) with a tensor processing unit. Evaluation is performed from 5000 to a maximum of 125,000 connected network nodes. To cover the maximum nodes in path, the test starts from node zero (x = 0, y = 0, z = 0) and ends on max assigned node. For the evaluation of path cost, every node is randomly assigned a cost between 0 to 10 in *x*, *y*, and *z* dimensions, and the same cost is used for every test.

### 6.1. Cost and Hops

**Figure 14:**
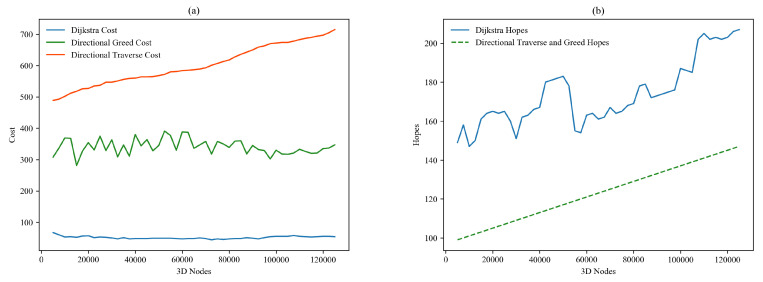
Cost and hops of the path in massive connected nodes in multilayer 6G hive.

Results of the cost and hops test of massively connected nodes are illustrated in Figure 14. Figure 14a demonstrates the total cost of paths from 5000 nodes to maximum of 125,000 nodes. Cost of 3D-Dijkstra remains under 100 throughout the test, and cost of directional traverse continuously spikes from 500 to 700, while directional greed remains in the middle with ups and downs between 275 to 375 in the continuous testing period. Figure 14b shows the maximum hops involved in every path. Directional traverse and directional greed algorithms show the behavior of linear diagonal with a continuous spike from 99 to 150 hops, and Dijkstra behaved differently on every path, but the number of hops remains much higher than directional algorithms.

**Figure 15:**
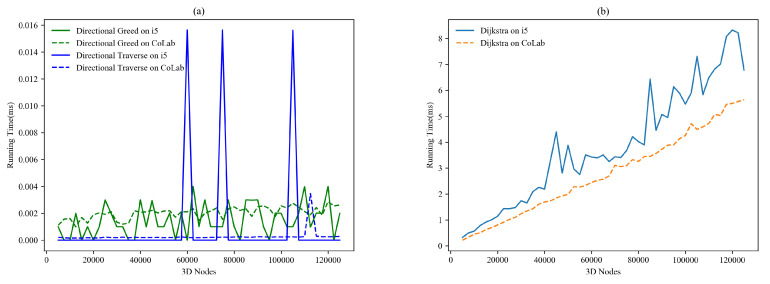
Running time of 3D pathfinding algorithms on i5 and CoLab.

Calculation of run time is the most important measure to judge the futuristic sustainability of a particular program. Figure 15a encompasses the run time of directional algorithms on i5 and CoLab; dashed lines represent the run time on CoLab hits, and straight lines represent i5 hits in milliseconds (ms). Response of directional traverse remains at 0 ms, and on i5 remains at 0 ms, except for three unexpected spikes up to 0.016 ms. Directional greed shows almost the same response on both devices, but behavior on i5 shows sharp ups and downs, and otherwise smooth around 0.002 ms. The run time of Dijkstra is far more than directional algorithms; as demonstrated in Figure 15b, it is continuously increasing with the increment of connected nodes. In CoLab, Dijkstra shows fewer bumpy spikes than its response on i5, and Dijkstra responds quicker than i5. The initially released code of these three algorithms is also provided on GitHub for reference [26].

### 6.2. Gains from Proposed Bee Hive Architecture

The distributed infrastructure of Bee Hive architecture provides numerous benefits, such as less traffic on a higher level of the network due to resources deployed on the ground. The town cell cloud deals with most of the traffic locally, which provides low latency and security from foreign objects. Furthermore, dynamic network slicing enhances security by isolation of different environments of the network. This section discusses the different properties of Bee Hive architecture and the benefits gained by those properties.

#### 6.2.1. AI and Physical Fog Implementation

The proposed Bee Hive architecture provides an on-door cloud; a local cell station works as a local cloud of the town cell. Partial AI application runs on smart devices such as cell phones, and the rest of the applications run on the cloud, which makes efficient execution of the application for smart devices having less computation power. All devices communicate with the local town cell cloud, which helps to save the overall bandwidth of the network and reduces packet loss. The cell station also controls the continuous connectivity of moving objects within the cell, and if the object moves out of the range of the town cell, then the smart cell tower pre-informs the neighbor cell tower to connect the incoming object. Pre-identification of object movement can easily be achieved through a pattern recognition technique, which is a sub-branch of AI and machine learning.

#### 6.2.2. Dynamic Network Slicing

Software-defined networking (SDN) and network function virtualization (NFV) are the core parts of the proposed Bee Hive architecture, which makes the 6G network resource-as-a-service (RaaS)-oriented. For better utilization of resources, idle networking hardware resources are divided into virtual slices. Multiple applications run parallel on slices through the implementation of virtualization on the local or central cloud. AI can play a vital role in the creation of different classes of the same nature devices that helps to create slices. MNOs can also create classes by given proprietary criteria according to their bylaws.

#### 6.2.3. Security

Bee Hive provides a distributed cloud approach, which divides the infrastructure into three layers: city cloud layer, town cell cloud layer, and edges layer with multiple smart devices. This approach speeds up the computation and adds an extra security layer between the main cloud and smart devices. We introduced SDN and NFV, which adds an extra strength to the security of data and connected devices [27]. Slicing algorithms were implemented in our local cloud to isolate the different types of users. Critical sectors that require isolation of services can take advantage of slicing such as banks and financial institutions. Blockchain can also be implemented to secure different distributed spectrums and slices of the network [28]. Distributed denial of service (DDoS) attacks are challenging for networks and clouds due to silent botnet. Bee Hive minimizes the threat of DDoS by traffic mining with certification of devices, and each task can easily check certificate credentials with minimum resources.

#### 6.2.4. Load Distribution

**Figure 16:**
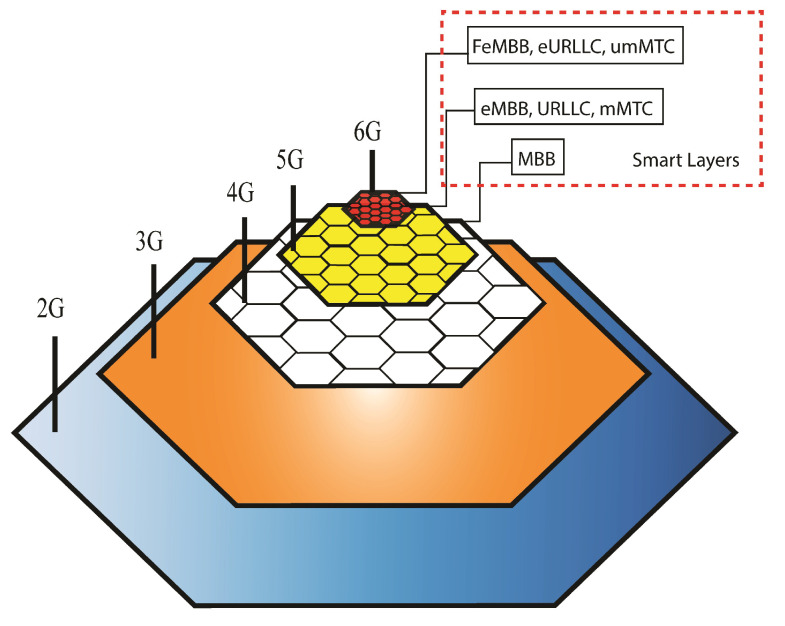
Network layers.

The traffic and data transmission load of the smart city is distributed in multiple town cells that relieve the upper network from massive traffic flow, and it helps to speed up the lower level traffic due to high computation power on the ground. This load distribution approach causes low latency of data transmission, due to the high bandwidth of 6G on the ground, and improves the throughput of the system by executing all algorithms of smart devices on the local cloud. Bee Hive architecture also provides a network layer system for low-data-consuming devices and for backward compatibility of previous generations, as shown in Figure 16. This network layer system ensures smooth traffic distribution through cell stations on previous generations such as 4G, 5G, and 6G. The city gateway can directly manage 3G and 2G; in case the size of the city area is larger than the coverage area of 2G and 3G, then extra resources can be plugged into selected cell stations for smooth transmission handling in the designated area.

The proposed Bee Hive architecture supports 6G traffic for full enhanced mobile broadband (FeMBB), extreme ultra-reliable low-latency communication (eURLLC), ultra-massive mobile-type communication (umMTC), and other communication types that have high bandwidth and low latency requirements. The 5G can be used for enhanced mobile broadband (eMBB), ultra-reliable low-latency communication (URLLC), and massive mobile-type communication. The 4G can be used as simple mobile broadband (MBB) and for backup of 5G and 6G in case of high traffic on these bands. The 3G and 2G can be used for long-distance communications. This distribution creates uniformity in the network and causes less load on each band, which avoids network overload and congestion.

#### 6.2.5. Support for Industrial Internet of Things

The 6G provides great communication wireless links for industrial IoT solutions and proposed Bee Hive local cloud services that can be utilized by industrial IoT objects. The town cell station cloud can be customized for the industrial area according to the requirement of the industry. Cloud resources can also be shared through different virtualization techniques, such as hypervisors and containers.

#### 6.2.6. Intelligent Radio Access

Bee Hive provides an intelligent radio (IR) for the support of 6G for ten times more dense connectivity of smart devices and large-scale AI implementation algorithms. The antenna size of 6G is shorter than 5G due to the short wavelength. For this purpose, the proposed Bee Hive architecture supports UM-MIMO instead of M-MIMO, which deals with dense device connectivity with the help of beamforming. The beamforming is based on multiple distributed queuing-based schemes such as non-orthogonal multiple access (NOMA), sparse code multiple access (SCMA), and delta orthogonal multiple access (D-OMA), which can help in connection with devices [29,30].

#### 6.2.7. Strong Backhaul

**Figure 17:**
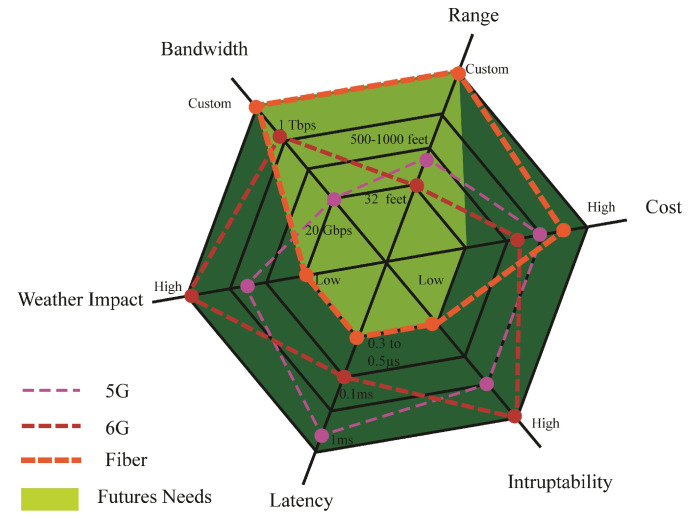
Backhaul links and future needs.

Bee Hive provides a strong fiber backhaul that acts as the backbone of the network. The backhaul link supports certain properties such as high bandwidth, high range, less weather allergy, low latency, and less interrupt ability. The 6G is the band of high bandwidth and low latency, but it can fail as backhaul due to less range and high electromagnetic interference through different solid objects and water. The 5G can have a supportive role, but in the case of backhaul, it cannot bear the load of the traffic. The 4G and previous generations are used for lightweight communications, but these generations cannot bear the weight of the smart world. Figure 17 elaborates that the fiber link is the most optimal link for future tendencies. Nested Bee Hive architecture provides self-legislation to use backhaul according to the density of the cell, but the town cell should be connected with a fiber link. For best QoS, the edge node of 6G should be connected entirely with a fiber link.

**Figure 18:**
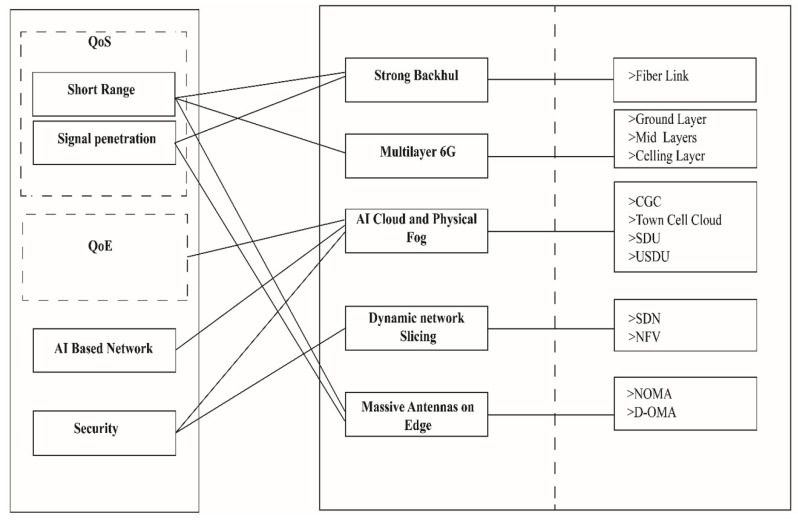
Major challenges resolved by Bee Hive.

Figure 18 demonstrates the major problems that the 6G network has to face in a smart environment and how the proposed Bee Hive architecture resolves these problems with different techniques. The short range of 6G is resolved with a massive antenna structure on the edge, a multilayer concept of 6G, and fiber backhaul. Signal penetration issue is the main cause of using fiber backhaul in Bee Hive, and a massive antenna on the edge reduces signal penetration by providing multiple alternative routes. QoE is resolved with the help of distributed AI cloud, which helps to improve user experience. AI-centric cloud and smart distribution units on the edge help to run multiple AI algorithms, which makes the Bee Hive network AI-centric. Certification of connected devices, distributed cloud infrastructure, and dynamic network slicing ensures the security of the Bee Hive network.

### 6.3. Obstacles and Future Directions

The Bee Hive architecture satisfies most of the expectations from the futuristic network of the smart city, but there are some extra expectations, such as ground-to-sky THz communication link and wireless backhaul communication. The space observatory project Atacama Large Millimeter/submillimeter Array (ALMA) creates hope for non-terrestrial communication in THz waves [31], but this project is deployed in the dry atmosphere of the Atacama Desert, and very few places on Earth have the same atmospheric tendencies [32]. Short range and atmospheric attenuation of THz waves are the biggest hindrances for wireless backhaul and non-terrestrial communication. Furthermore, the massive structure of 6G in streets will be costly and may cause public resistance due to fear of electromagnetic radiation. Further obstacles and future directions to overcome those obstacles are discussed in Table 1.

## 7. Conclusions

Overpopulation is the core of problems in urban areas; the capacity of our megacities is overflowing, and the situation will become worse in the upcoming future. A smart management system such as a smart city is needed to resolve these issues. The idea of the smart city with 5G might spare us a decade, but after that, a smart city needs to be upgraded to its next generation with 6G and AI. The 6G is expected to be revolutionary in the wireless network domain, but it has short range and moisture-related properties, which may cause complications in long- and even medium-distance communication. This study proposes a low-latency, high-bandwidth, AI-centric, and futuristic multilayer physical network architecture of 6G for futuristic smart cities. Nested Bee Hive architecture provides an on-ground cloud network that helps smart devices to run AI applications partially on their own and the rest on the cloud. Furthermore, the distributed and edge computing-oriented infrastructure of Bee Hive provides security and reduces traffic load on the upper layer of the network. Bee Hive handles all previous generations consecutively, which provides traffic distribution leverage on a different spectrum according to data usage requirements, and provides backup to 6G in lousy weather. The Bee Hive provides a pluggable pathfinding module which provides an easy upgradation according to the need of the hive. The technology is snowballing continuously; thus, it is too early to define final paradigms for the future of 6G in futuristic smart cities, but Bee Hive provides a perfect initial pattern to move forward. Bee Hive provides futuristic luxuries to the smart city but it is an expensive facility due to dense connectivity and massive resources deployment on the edge. Thus, in the future, there is a need for cost reduction and the upgradation of communication links and other essential hardware.

## Figures and Tables

**Table 1:** Obstacles for 6G and Bee Hive.

Obstacles	Description	Future Directions
Wireless Backhaul	Massive data-carriage capacity of THz waves creates aspiration of wireless backhaul in futuristic network, but THz waves are short-range and highly allergic to materials and moisture; thus, these waves cannot be used as backhaul.	Further research needs to be carried out in wireless backhauling technique to identify cheap, low-latency, less interruptive, long-range, and massive bandwidth backhaul.
Non-Terrestrial Communication	A chain of mediums is required to be deployed in different layers of Earth’s orbit, but the real problem is in high gravitational pull area below 350 km altitude. Flying objects remain under the Karman line within 90 km.	High-altitude flying aircrafts can be deployed with special communication antennas and high computation power to interlink with satellite [33,34]. For now, it is a vague theory without any concrete proof; further research is required to implement this hypothesis of non-terrestrial network (NTN).
High Cost	Bee Hive is an expensive facility due to massive resource deployment on edge and overhead of fiber connectivity. For the deployment of non- terrestrial network, 6G is too expensive and also not economical for terrestrial networks.	Research for new cheap and effective hardware and communication links should be undertaken to reduce the cost of necessary equipment for TN and NTN.
Health Hazards	Biological researchers have some concerns about the outcomes of continuous exposure to electromagnetic waves [35,36]. Low-power distributed antenna structure reduces the concerns of skin burn and DNA damage [37,38]. However, a little incident from THz frequency can escalate the technophobic mist.	There is a need for further research in the standardization of 6G bands, which are safe to use for humans and other natural objects.

## Data Availability

Not Applicable.
